# Comparison of the fecal microbiota of two free-ranging Chinese subspecies of the leopard (*Panthera pardus*) using high-throughput sequencing

**DOI:** 10.7717/peerj.6684

**Published:** 2019-03-28

**Authors:** Siyu Han, Yu Guan, Hailong Dou, Haitao Yang, Meng Yao, Jianping Ge, Limin Feng

**Affiliations:** 1 Northeast Tiger and Leopard Biodiversity National Observation and Research Station, Ministry of Education Key Laboratory for Biodiversity Science and Ecological Engineering, State Forestry and Grassland Administration Key Laboratory for Conservation Ecology of Northeast Tiger and Leopard National Park, State Forestry and Grassland Administration Amur tiger and Amur leopard Monitoring and Research Center, College of Life Science, Beijing Normal University, Beijing, China; 2 College of Life Sciences, Qufu Normal University, Shandong, China

**Keywords:** Amur leopard, Gut microbiota, Non-invasive, 16s rRNA gene, High-throughput sequencing, North Chinese leopard, Free-ranging leopard in China, Leopard conservation

## Abstract

The analysis of gut microbiota using fecal samples provides a non-invasive approach to understand the complex interactions between host species and their intestinal bacterial community. However, information on gut microbiota for wild endangered carnivores is scarce. The goal of this study was to describe the gut microbiota of two leopard subspecies, the Amur leopard (*Panthera pardus orientalis*) and North Chinese leopard (*Panthera pardus japonensis*). Fecal samples from the Amur leopard (*n* = 8) and North Chinese leopard (*n* = 13) were collected in Northeast Tiger and Leopard National Park and Shanxi Tieqiaoshan Provincial Nature Reserve in China, respectively. The gut microbiota of leopards was analyzed via high-throughput sequencing of the V3–V4 region of bacterial 16S rRNA gene using the Life Ion S5™ XL platform. A total of 1,413,825 clean reads representing 4,203 operational taxonomic units (OTUs) were detected. For Amur leopard samples, *Firmicutes* (78.4%) was the dominant phylum, followed by *Proteobacteria* (9.6%) and *Actinobacteria* (7.6%). And for the North Chinese leopard, *Firmicutes* (68.6%), *Actinobacteria* (11.6%) and *Fusobacteria* (6.4%) were the most predominant phyla. *Clostridiales* was the most diverse bacterial order with 37.9% for Amur leopard and 45.7% for North Chinese leopard. Based on the beta-diversity analysis, no significant difference was found in the bacterial community composition between the Amur leopard and North Chinese leopard samples. The current study provides the initial data about the composition and structure of the gut microbiota for wild Amur leopards and North Chinese leopards, and has laid the foundation for further investigations of the health, dietary preferences and physiological regulation of leopards.

## Introduction

Leopards (*Panthera pardus*) are currently the most widely distributed wild felids (Jacobson et al., 2016), but they are confronted with worldwide population declines due to illegal poaching, prey depletion, habitat fragmentation, and anthropogenic disturbances (Balme, Slotow & Hunter, 2009; Hebblewhite et al., 2011; Kissui, 2008; Nowell & Jackson, 1996; Packer et al., 2011; Stein et al., 2016; Sunquist & Sunquist, 2002). The International Union for Conservation of Nature (IUCN) recognizes nine subspecies of leopards, including the Amur leopard and the North Chinese leopard (Miththapala, Seidensticker & O’Brien, 1996; Uphyrkina et al., 2001). The Amur leopard has been classified as critically endangered by IUCN since 1996 (Jackson & Nowell, 2008). Once patrolling from Northeast China to southernmost portions of the Russian Far East and the Korean peninsula (Nowell & Jackson, 1996), the Amur leopard is currently confined to the adjacent habitats in the Jilin and Heilongjiang provinces in China and southwestern Primorsky Krai in Russia (Feng et al., 2017; Hebblewhite et al., 2011). The North Chinese leopard originally distributed North and Central China but lost as much as 98% of their historic range. An accurate distribution area and population size still remain unclear due to the lack of empirical investigation (Jacobson et al., 2016). Recently, the Cat Classification Task Force of the IUCN Cat Specialist Group revised the taxonomy of leopards and included the North Chinese leopard in Amur leopard on account of the obscure biogeographical barrier between them (Kitchener et al., 2017), although North Chinese leopard was described as the typical subspecies in North China since 1862 (Allen, 1938; Gray, 1862). Moreover, other evidence based on molecular biology supporting this classification for the two leopards are scarce, especially in North China.

Amur Leopard and North Chinese leopard are large-sized feline species and solitary predators that play pivotal roles in the ecosystems where they occur. Many efforts have been made to uncover their dietary habits, population genetic structure, and individual identification for conservation purposes through non-invasive sampling of feces (Dutta & Seidensticker, 2013; Dutta et al., 2012; Rodgers & Janečka, 2013; Yang et al., 2018). Gut microbial diversity analyses based on leopard fecal samples should also be considered as an important part of conservation efforts. In-depth understanding of the relationship between host habitat and microbiota composition may be helpful for conservation efforts because changes in the gut bacterial communities have been shown to affect host metabolism and energy homeostasis (Musso, Gambino & Cassader, 2010).

The gut microbiota composition is an indicator of health condition for endangered wild animals, since habitat degradation may affect host health negatively via diet-associated shifts in the gut microbiota (Amato et al., 2013). Dietary changes caused by human disturbance and habitat degradation likely result in a decrease in microbiota diversity (Barelli et al., 2015). Animal groups from habitat under increased anthropogenic pressure could be distinguished by the comparison of gut bacterial communities (Gomez et al., 2015). Therefore, changes in the gut microbiota species composition of endangered animals might be used as an indicator of habitat degradation and fragmentation (Barelli et al., 2015).

Additionally, it has been shown that the detection of pathogenic bacteria is indicative of severe infectious diseases in endangered species (Zhao et al., 2017), and fecal bacterial composition could alter accordingly with gastrointestinal diseases in animals (Suchodolski et al., 2012). Research has shown that the fecal bacterial species richness was decreased and various bacterial taxa were altered in cats with diarrhea (Suchodolski et al., 2015). Compared with healthy cats, cats with clinical signs of gastrointestinal tract disease had significantly lower amount of microaerophilic bacteria (Johnston et al., 2001).

Residential gut bacteria are also able to serve as a natural barrier against invasive pathogens (Gibson et al., 1995), and to facilitate the function of the immune system (Maynard et al., 2012; Round & Mazmanian, 2009). Specific compositions of the gut microbiota are associated with variations in the host diet, phylogeny, and physiological status (Benson et al., 2010; De et al., 2010; Nelson et al., 2013; Sommer & Bäckhed, 2013). Characterization of gut bacterial communities is important in understanding the mechanisms of host–microorganism interactions (Nicholson et al., 2012). Thus, the gut microbiota analysis is fundamental and of paramount importance to the conservation of endangered species. Potentially, the studies of the gut microbiota could be an assistant tool for understanding the phylogenetic relationship of leopard subspecies in the future.

Here, we characterized and compared the fecal bacterial communities of the Amur leopard and North Chinese leopard via high-throughput sequencing targeting the V3–V4 hypervariable region of the bacterial 16S rRNA gene, and provide the first benchmark of gut bacterial diversity in Amur and North Chinese leopard that potentially contribute to further conservation research.

## Materials and Methods

### Sample collection

Opportunistic fecal sampling occurred in the period from December 2016 to March 2017 in the two distribution areas from the leopards. A total of eight (O1-O8) fecal samples of the Amur leopard were obtained from the Northeast Tiger and Leopard National Park located in the Heilongjiang and Jilin provinces of China (E129°05′–131°18′, N42°37′–44°10′). This distribution area of the Amur leopard is characterized by a monsoon climate with cold and windy winters, the main vegetation types are mixed broad-leaved forests and secondary Mongolian oak (*Quercus mongolica Fisch. ex Ledeb*) forest (Tian et al., 2015). A total of 13 fecal samples (J1-J13) of the North Chinese leopard were collected from Tieqiaoshan Provincial Nature Reserve in Shanxi province (E111°25′–114°17′, N36°39′–38°06′). This region belongs to the warm temperate continental climate with little snow in winter and dry wind in spring, and the vegetation forms are temperate deciduous broad-leaved forests (Zheng et al., 2009). Field experiments were approved by the Forestry Department of Jilin Province, State Forestry Administration and Forestry Department of Shanxi Province.

All fecal samples were collected simultaneously by several groups of our team in different sites. We designed line transects that leopards regularly used based on camera trapping data and sent trained members in field soon after snowfall to collect feces above snow layer where leopard footprint traces were present. Each line transect has been revisited more than once at a three day interval, only newly-excreted feces after previous inspection were collected with the sterile tools. The low environmental temperature below 0 °C contributed to the preservation of gut microbes in the fecal samples. Samples were stored in special ice boxes under −20 °C during in-field study and finally stored under −80 °C in laboratory for further experiments.

### DNA extraction

Total bacterial genomic DNA was extracted from fecal samples using QIAamp^®^ Stool Mini Kit (Qiagen, Hilden, Germany) following the manufacturer’s protocol. DNA quantity and quality were examined using NanoDrop™ One (Thermo Fisher Scientific, Waltham, MA, USA) according to the manufacturer’s instruction.

### Bacterial 16S rRNA genes amplification and sequencing

The V3–V4 hypervariable region of the 16S rRNA gene was amplified using primers 341F (5′-CCTAYGGGRBGCASCAG-3′) and 806R (5′-GGACTACNNGGGTATCTAAT-3′). PCR amplifications were conducted in a total volume of 50 µL mixture containing 6 µL of the template DNA, 25 µL of 2 ×Taq PCR Master Mix (0.1 U/µL; KHBE, China), 2 µL of each primer (10 µM) and 15 µL ddH_2_O. The reaction system was then subjected to 1 cycle of initial denaturation at 95 °C for 3 min, followed by 25 cycles at 95 °C for 30 s, annealing at 55 °C for 30 s and extension at 72 °C for 30 s, and a final cycle at 72 °C for 5 min. Stained with SYBR^®^ Safe DNA Gel Stain (Invitrogen, Carlsbad, CA, USA), the PCR products were assessed using electrophoresis in 2% agarose gels and visualized under UV light. The PCR products were purified using the GeneJET (Thermo Fisher Scientific, Waltham, MA, USA).

The sequencing libraries were generated using Ion Plus Fragment Library Kit 48 rxns (Thermo Fisher Scientific, Waltham, MA, USA). DNA concentrations of PCR products were quantified through Qubit and subjected to quality control procedures (Edgar et al., 2011; Haas et al., 2011; Martin, 2011). High-throughput sequencing was performed on a Life Ion S5TM XL (Thermo Fisher Scientific, Waltham, MA, USA) following the manufacturer’s instructions.

The data set of our study is available in the Sequencing Read Archive (SRA) on NCBI with accession numbers of SRP149194.

### Sequence processing and data analysis

The original sequencing reads were trimmed using Cutadapt V1.9.1 (http://cutadapt.readthedocs.io/en/stable/) (Martin, 2011). Raw reads were obtained after removing barcode and primers. Chimeric sequences were checked and eliminated based on UCHIME Algorithm (http://www.drive5.com/usearch/manual/uchime_algo.html) (Edgar et al., 2011) and Gold database (http://drive5.com/uchime/uchime_download.html) in order to generate clean reads.

For all samples, OTUs were generated from clean reads via Uparse v7.0.1001 software (http://drive5.com/uparse/) with a 97% sequence identity cutoff value (Edgar, 2013). Using Mothur (Schloss et al., 2009), representative sequences of the OTUs which were chosen by the highest frequency of occurrence, were annotated against the SILVA SSUrRNA database (http://www.arb-silva.de/) (Quast et al., 2013; Schloss et al., 2009; Wang et al., 2007) and aligned by MUSCLE (Version 3.8.31) (Edgar, 2004) to construct the phylogenetic relationship between different OTUs.

Series of alpha-diversity indices including Observed species, Shannon, Simpson, Chao1, ACE, and Goods coverage were calculated and analyzed in QIIME (Version 1.9.1) (Caporaso et al., 2010). The rarefaction curves and rank abundance curves were constructed in R (Version 2.15.3). We applied Wilcoxon rank-sum test to identify discrepancies of gut bacterial diversities between the Amur leopard and North Chinese leopard for each index of alpha-diversity.

Using the QIIME pipeline (Version 1.9.1), beta-diversity was assessed by calculation of Unifrac distances and subsequently visualized by principal component analysis (PCoA). Phylogenetic trees were also built using UPGMA (unweighted pair-group method with arithmetic mean). The principal component analysis (PCA), principal co-ordinate analysis (PCoA) and non-metric multidimensional scaling (NMDS) were calculated using R (version 2.15.3) (R Core Team, 2013) so as to evaluate the similarity and discrepancies of bacterial communities among fecal samples based on weighted and unweighted distance matrix. The Analysis of Similarities (ANOSIM) was also used to testify whether there was a significant difference between two groups (Clarke, 1993). Beta-diversity was then subjected to Wilcoxon rank-sum test.

## Results

### Overall sequencing data

A total of 1,514,233 raw reads were yielded after high-throughput sequencing of all samples. The data sets were then subjected to quality control procedures which resulted in 1,413,825 clean reads for the 21 samples analyzed. The total number of OTUs was 4,203 at a threshold of 97% sequence identity for all samples.

**Figure 1:**
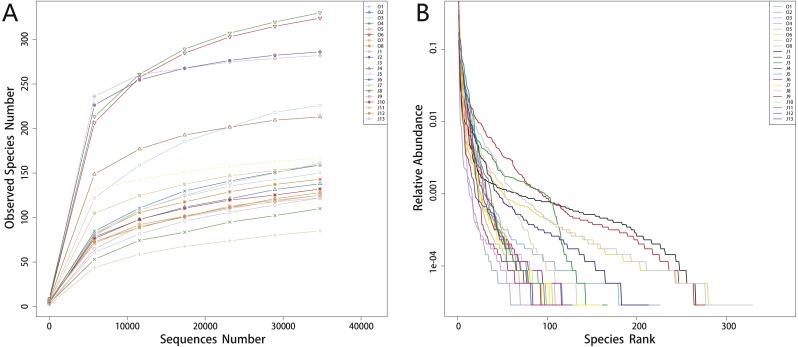
Rarefaction curves (A) and rank abundance curves (B). The *x*-axis of rarefaction curves indicates the sequences number selected randomly from samples and the *y*-axis indicates the observed species number (OTUs). The curves in (A) tend to be flat reflect that the sequencing data size is rational. In rank abundance curves (B), the *x*-axis indicates the order number ranked by the OTUs abundance while *y*-axis shows the relative abundance of OTUs. The higher the richness of the species, the larger the span of the curve on the horizontal axis. In the vertical direction, the smoother the curve, the more uniform the species distribution.

Alpha-diversity indices including Observed species, Shannon, Simpson, Chao1, ACE and Goods coverage are shown in Table 1. The rarefaction curves showed a pattern of plateau formation (Fig. 1A), indicating that the microbial diversity present in each sample was sufficiently quantified at this sequencing depth. We also analyzed the rank abundance curves to evaluate the abundance and distribution of bacteria taxa (Fig. 1B).

**Table 1:** Alpha-diversity of fecal microbiota in Amur leopard and North Chinese leopard feces.

**Sample**	**Observed species**	**Shannon**	**Simpson**	**Chao1**	**ACE**	**Goods coverage**
O1	226	3.205	0.788	248.521	258.106	0.999
O2	138	2.959	0.747	173.455	190.370	0.999
O3	85	0.683	0.146	108.400	117.380	0.999
O4	110	2.137	0.649	161.250	163.516	0.999
O5	125	1.704	0.433	144.077	157.104	0.999
O6	324	4.183	0.867	350.757	351.019	0.999
O7	159	3.875	0.85	173.040	181.306	0.999
O8	143	3.736	0.867	172.750	174.050	0.999
J1	282	3.489	0.666	294.364	289.486	1.000
J2	286	5.798	0.961	300.056	298.907	0.999
J3	167	4.412	0.855	194.273	195.119	0.999
J4	213	4.086	0.868	237.474	233.225	0.999
J5	150	3.871	0.888	170.036	178.518	0.999
J6	159	3.128	0.786	202.000	203.286	0.999
J7	161	2.563	0.722	246.550	234.682	0.998
J8	330	4.813	0.909	370.886	364.072	0.998
J9	125	3.454	0.854	151.400	160.056	0.999
J10	132	2.946	0.791	145.500	156.117	0.999
J11	122	4.169	0.919	139.105	146.006	0.999
J12	128	3.473	0.853	308.167	201.223	0.999
J13	122	1.395	0.333	157.286	164.553	0.999

### Bacteria composition and relative abundance

Overall, we identified 28 phyla, 55 classes, 88 orders, 167 families and 344 genera of bacteria in the gut microbiota community from 21 fecal samples of leopards.

**Figure 2:**
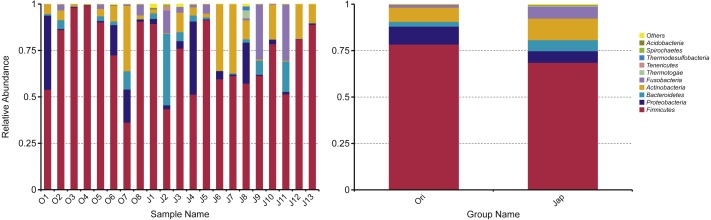
Fecal bacterial composition of Amur leopard and North Chinese leopard at the phylum level. The top ten bacterial phyla chosen according to the results of species annotations were ranked by the relative abundance in each sample or group. The *x*-axis and *y*-axis represents the information of samples and relative abundance respectively.

For the Amur leopard, *Firmicutes* was the predominant phylum (78.4%) (Fig. 2). *Proteobacteria* (9.6%), *Actinobacteria* (7.6%), *Bacteroidetes* (2.6%) and *Fusobacteria* (1.7%) contributed also to the total composition. At the family level, *Planococcaceae* (30.1%), *Clostridiaceae 1* (17.2%) and *Peptostreptococcaceae* (14.5%) were the top 3 dominant families. At the genus level, *Sporosarcina* was predominant with an abundance of 22.8%, followed by *Clostridium sensu stricto 1* (17.1%) and *Peptoclostridium* (10.2%).

For the North Chinese leopard, *Firmicutes* (68.6%) was the most predominant phylum (Fig. 2), followed by *Actinobacteria* (11.6%), *Fusobacteria* (6.4%), *Proteobacteria* (6.2%) and _*Bacteroidetes* _(6.0%). *Clostridiaceae_1* (19.5%), *Planococcaceae* (16.2%) and *Lachnospiraceae* (12.5%) were the three most predominant families. At the genus level, *Clostridium sensu stricto 1* (19.4%), *Sporosarcina* (9.5%) and *Peptoclostridium* (6.1%) constituted the top three genera.

**Figure 3:**
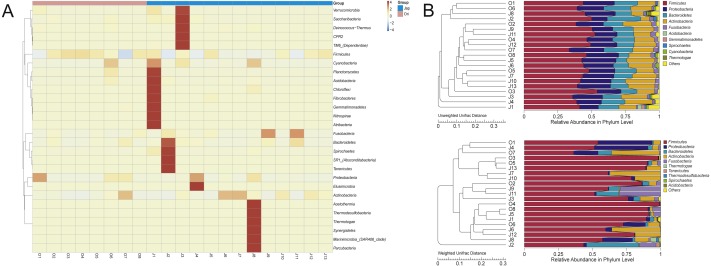
The heatmap of clustering for species richness (A) and UPGMA clustering trees with relative abuandance in phylum level (B). The heatmap of clustering for species richness (A) illustrates the bacterial distribution among different fecal samples of leopards. The bacterial phyla were clustered for their relative abundance. The *x*-axis represents each sample, and the *y*-axis represents the relative percentage of each bacterial phyla. The *Z*-value (−4 to 4), displayed by color intensity, is the relative abundance of the sample and the difference in the average relative abundance of all samples divided by the standard deviation of all samples in the classification. In (B), the UPGMA clustering trees were generated with the weighted and unweighted Unifrac distance and then we integrated the trees with the relative abundance of species among all samples in phylum level. The relative abundance of species at phylum changed as the main composition phyla changed based on different Unifrac distance.

The clustered heatmap showed (Fig. 3A) that the gut bacterial distribution of the Amur leopard and North Chinese leopard were relatively scattered. The unweighted pair-group method with arithmetic means (UPGMA) (Fig. 3B) that display the similarities between sample groups showed a similar result with the clustered heatmap.

### Differences in community composition

**Figure 4:**
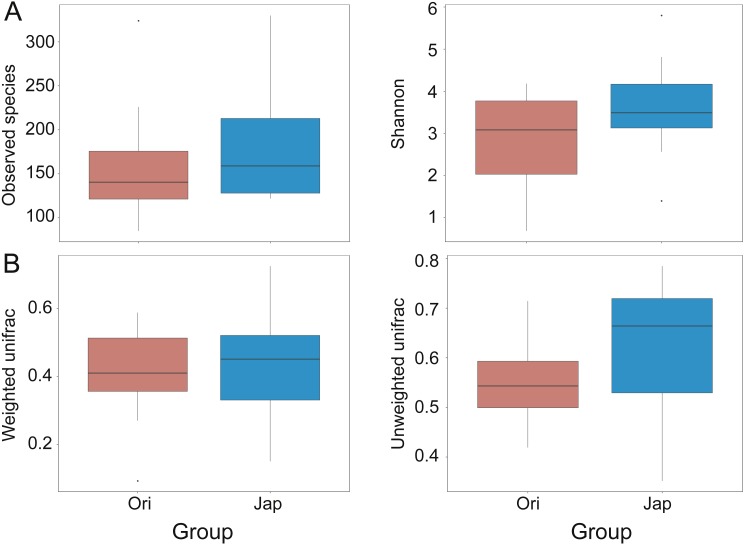
Comparisons of alpha (observed species and Shannon index) (A), and beta-diversity (with weighted and unweighted Unifrac distance matrix) (B), between Amur leopard and North Chinese leopard fecal samples.

The boxplots of alpha diversity were shown in Fig. 4A. Observed species and Shannon diversity indices were tested for the significance of discrepancies between the two sample groups (*p* = 0.447 and 0.210, respectively). The beta-diversity indices were presented in Fig. 4B (*p* = 0.441 and 0.003, respectively), illustrating the discrepancies of gut bacterial communities between different groups. The Analysis of Similarities (ANOSIM) showed the significance level between different groups (*R* = 0.02, *p* = 0.335) (Fig. S1). The heatmap of beta-diversity indices were also plotted in Fig. S2.

**Figure 5:**
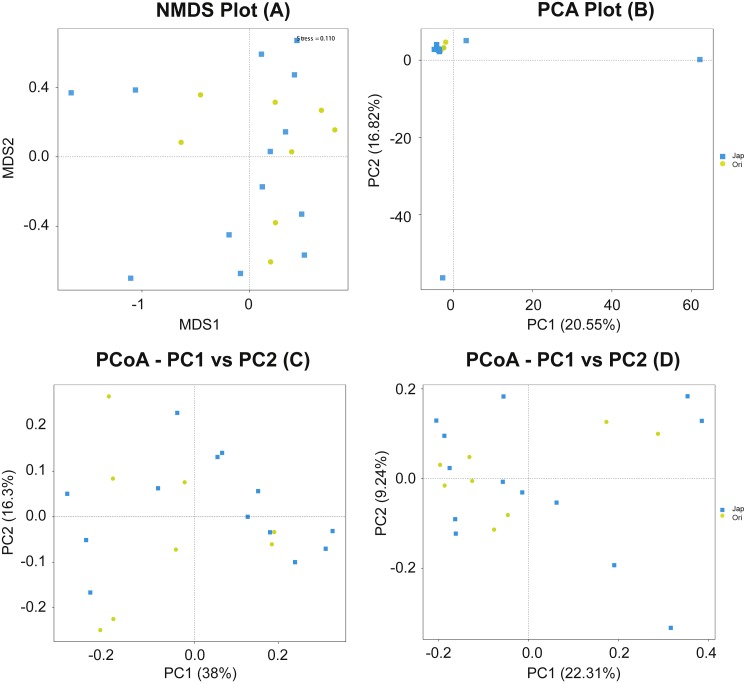
NMDS (A), PCA (B) and PCoA (C & D) of fecal bacterial community structures of Amur leopard and North Chinese leopard. The yellow points represent Amur leopard and the blue squares represent North Chinese leopard. For PCoA (C) and (D) were analyzed with weighted Unifrac distance and unweighted Unifrac distance respectively. All the points are scattered, which indicates that no significant differences were found between the two subspecies.

Non-metric multidimensional scaling (NMDS) displayed separation in gut microbiota composition of the Amur and North Chinese leopard, and the stress value less than 0.2 could show the discrepancy between samples was 0.110 (Fig. 5A). The PCA plot (Fig. 5B) revealed that the samples from the two subspecies were basically clustered together. The main components of the gut microbiota of the Amur leopard and North Chinese leopard were similar with two exceptional samples from the North Chinese leopard. According to the PCoA analysis (Figs. 5C and 5D), the fecal bacterial communities of Amur leopard and North Chinese leopard were relatively scattered in every group. Overall, no significant differences were found between the two sample species according to the results of PCoA, PCA and NMDS.

## Discussion

With the rapid development of high-throughput sequencing technology, there are mounting studies focusing on the analysis of gut microbiota in different vertebrates. Amur leopards and North Chinese leopards are endangered flagship species facing severe survival predicament result from prey depletion, habitat fragmentation, and anthropogenic disturbances. However, research effort on free-ranging leopards in China, especially for North Chinese leopards are neglected (Jacobson et al., 2016). In this study, we characterized and compared the gut microbiota of Amur leopards and North Chinese leopards for the first time using high-throughput sequencing technology. The characterization of their gut microbiota might be able to provide useful information for potential research and help us evaluate the healthy condition of wild leopards in their natural habitat.

Five major bacterial phyla were observed including *Firmicutes*, *Proteobacteria*, *Actinobacteria*, *Bacteroidetes* and *Fusobacteria* both in the Amur leopard and North Chinese leopard samples, which is in accordance with the vertebrate gut microbial diversity described by many other studies (Deng & Swanson, 2014; Ley et al., 2008; Ritchie, Steiner & Suchodolski, 2008). Fecal samples of healthy cats are featured with similar phylum composition with slightly different proportions (Barry et al., 2012). Based on our analysis, no significant difference was found in the relative abundance of these five phyla between the samples from the Amur leopard and North Chinese leopard.

*Firmicutes* was the most predominant phylum in both the Amur leopard and North Chinese leopard and showed no significant difference between two groups (*p* = 0.210). Previous researches have reported that *Firmicutes* is the most dominant phylum in feces of animals (Garcia-Mazcorro et al., 2012; Guan et al., 2017; Ritchie et al., 2010) and humans (Arumugam et al., 2011). Same tendency was also found in feline species in the wild such as leopard cats (*Prionailurus bengalensis*) (An et al., 2017) and snow leopards (*Panthera uncia*) (Zhang et al., 2015). Some studies reported that the body fat storage influences the gut bacterial composition in mice (Ley et al., 2005) and humans (Ley, Peterson & Gordon, 2006). A significantly greater proportion of *Firmicutes* and a significant reduction of *Bacteroidetes* were observed in obese animals than in lean controls (Turnbaugh et al., 2006). The tendency of an increase in *Firmicutes* and a decrease in *Bacteroidetes* was associated with switching to the high fat diet (Hildebrandt et al., 2009; Tremaroli & Bäckhed, 2012). We detected that the proportion of *Firmicutes* in Amur leopards was relatively greater than in North Chinese leopards, and the proportion of *Bacteroidetes* in Amur leopards was relatively lower which indicated that the weight of Amur leopard should be more heavier. This might relate to the greater body fat storage of Amur leopards compare with North Chinese leopards, since Amur leopards have larger body size and store more fat to withstand severe cold in further north habitat (Wang et al., 2017). Unfortunately, the detail information about wild North Chinese leopard is comparatively scarce.

Within the phylum *Firmicutes*, Zhang et al. (2015) found that *Lachnospiraceae* was the most diverse family in the feces of snow leopards, which is consistent with a previous report in wolves (*Canis lupus*) (Zhang & Chen, 2010). In our results, however, the most diverse family was *Clostridiaceae 1* (19.5% in North Chinese leopard, 17.2% in Amur leopard) within the order *Clostridiales*, and *Lachnospiraceae* constituted a relatively small proportion in our sample set compared to snow leopards and wolves. *Lachnospiraceae* was found in both human and mammal gut microbiota that relates to some diseases like colon cancer (Meehan & Beiko, 2014), nonalcoholic fatty liver disease (NAFLD) (Shen et al., 2017) and diabetes (Kameyama & Itoh, 2014). However, without sufficient support based on other health monitoring methods including blood or apparatus test, the proportion of *Lachnospiraceae* in the gut microbiota could only be a simple referential marker that reflects health condition for wild animals.

Our results also indicated that *Clostridium sensu stricto 1* was a predominant genus in the gut microbiota of leopards. And *Clostridium perfringens* was a common bacterial species for both the Amur leopard and North Chinese leopard. Lubbs et al. (2009) reported that the gut microbiota of domestic cats is affected by the protein concentration in diets, particularly, *Clostridium* populations increased as more protein was digested. The presence of *C. perfringens* was positively associated with protein intake in grizzly bears (*Ursus arctos*) (Schwab et al., 2011) and cheetahs (*Acinonyx jubatus*) (Becker et al., 2014). To our knowledge, leopards are highly carnivorous and consume mostly protein in their daily diet (Martins et al., 2011). We speculate that the high proportions of *Clostridium* populations might reflect the high-protein diet of leopards in our study. Interestingly, *C. perfringens* might be potential pathogenic bacteria that cause diarrhea in dogs (*Canis lupus familiaris*) and cats (*Felis catus*) (Suchodolski, 2011). However, *C. perfringens* was also detected in the clinically healthy dogs and house cats (Handl et al., 2011; Queen, Marks & Farver, 2012). *C. perfringens* should probably be considered as a common commensal in the intestine of healthy feline (Becker et al., 2014).

The relationship between gut microbiota and gastrointestinal diseases including inflammatory bowel disease (IBD), chronic enteropathies (CE), and acute diarrhea in Carnivora are well- documented (Suchodolski, 2016). For examples, there are increases in the proportions of bacterial genera belonging to *Proteobacteria* and decreases in *Fusobacteria*, *Bacteroidetes*, and *Firmicutes* in canine IBD (Yasushi et al., 2015). And an increase of *Enterobacteriaceae* along with decreased proportions of *Bacteroidetes*, *Faecalibacterium* spp. and *Turicibacter* spp. were observed in cats with chronic diarrhea (Suchodolski et al., 2015). It indicates that some gut microbiota dysbiosis, which caused by disease processes, can be identified in fecal samples (Suchodolski, 2016).

*Proteobacteria* was another phylum in the gut microbiota of the leopards and was not significantly different between two subspecies (*p* = 0.804). *Proteobacteria* was also detected in other feline gut microbiota analysis based on different methods (Ozaki et al., 2009; Ritchie, Steiner & Suchodolski, 2008). As the most predominant phylum in giant panda (*Ailuropoda melanoleuca*), *Proteobacteria* play crucial role in degrading lignin, which is the main ingredient of bamboo (Fang et al., 2012), and in catabolizing complicated compounds in fodder (Evans et al., 2011). *Proteobacteria* was the most predominant phylum in the gut microbiota of dogs in the obese groups while in the lean groups was *Firmicutes* (Park et al., 2015), and the proportion of *Proteobacteria* was related with inflammatory bowel disorder (IBD) and *Clostridium difficile* infection (Chang et al., 2008; Packey & Sartor, 2009) as well. For many large mammals in the wild, noninvasive sampling such as collecting feces or hairs are relatively feasible and effective method to obtain information, but the real-time living situation and health condition of some wild species are still unclear, as well as the definite function of different bacteria in host health.

The phylum *Actinobacteria* also contributed to the gut microbiota of the leopards and its proportion was not significantly different between the two subspecies (*p* = 0.414). It was identified to be the most predominant phylum in snow leopards (Zhang et al., 2015). In contrast, Wu et al. (2017) reported that *Actinobacteria* constituted 0.53% of all gut bacteria in wolves (*Canis lupus*). Handl et al. (2011) found that *Actinobacteria* constituted 7.3% of all bacterial sequences in house cats, but was at a low abundance in dogs (1.8%). This result in regard to dogs was in line with an analysis using 454 pyrosequencing (Middelbos et al., 2010). There might be a different tendency in the abundance of *Actinobacteria* between feline and canine species, or perhaps the abundance of *Actinobacteria* in the mammalian gut is currently biased, because sequencing methodology without prior %G+C profiling might underestimate the proportion of high G+C bacteria including *Actinobacteria* (Harri et al., 2009).

*Bacteroidetes* was another contributive phylum in the gut microbiota of the leopards and showed no significant difference between groups (*p* = 0.707). *Bacteroidetes* ranks over *Firmicutes* as the most predominant phylum in some cases, as shown in domestic cats and dholes (*Cuon alpinus*) (Jacobson et al., 2016; Tun et al., 2012). The relative abundance of *Bacteroidetes* varied significantly in different studies (Handl et al., 2011; Ritchie et al., 2010). Within *Bacteroidetes*, the genus of *Bacteroides* contributed 1.1% and 3.2% to the gut microbiota of the Amur leopard and North Chinese leopard, respectively. *Bacteroides* species were reported that took part closely in the breakdown of complex molecules, such as polysaccharides, also the biotransformation of bile acids (Lan et al., 2006; Reeves, Wang & Salyers, 1997). Additionally, this crucial genus could inhibit some pathogenic micro-organisms (like *Escherichia coli*, *Klebsiella pneumonia*) with other anaerobic bacteria, which beneficial to host (Hentges, 1983; Van der Waaij, Berghuis-de Vries, 1971 & Lekkerkerk-Van der Wees). Although the presence of *Bacteroides* in the gut microbiota might be beneficial to the health condition of leopard to some extent, more feces samples should be collected for further investigation with camera trap data and identified individual in the nature reserves to prove the above inferences.

With regard to other phylum *Fusobacteria* detected in the feces of leopards, no significant difference was found between samples from the two subspecies (*p* = 1.000). Research based on pyrosequencing suggested that *Fusobacteria* were less abundant in domestic cats than in domestic dogs (Garciamazcorro et al., 2011). This tendency might be analogous to the relationship of gut microbiota observed in raccoon dogs (*Nyctereutes procyonoides*) and leopard cats (An et al., 2017).

In summary, although the proportions of the five predominant bacterial phyla are slightly different among the gut bacteria of the Amur leopard and North Chinese leopard, no significant difference was found in phylum composition between the two subspecies. Previous work has shown that the gut microbial community structure of species can vary in different environments (Clayton et al., 2016), and respond to dietary alterations, including the amount and type of dietary fiber or other bioactive food components (Turnbaugh et al., 2008). The potential prey for the Amur leopard includes Siberian roe deer (*Capreolus pygarus*), sika deer (*Cervus nippon*), wild boar (*Sus scrofa*) and Badger (*Meles meles*) in the Northeast Tiger and Leopard National Park (Yang et al., 2018). However, sika deer is not available for North Chinese leopard in Tieqiaoshan Provincial Nature Reserve (Wu, Wan & Fang, 2004), which indicates that in addition to the relative different dietary components and living environment, there are other influence factors play crucial roles for the gut microbiota of wild leopard. We speculate that genetic factors might be responsible for the same tendency in gut microbiota composition, after all the classification of this two subspecies leopard is still controversial around the world due to the lack of efficient evidence in molecular biology. At the genus level, however, the bacterial composition for each fecal sample is individualized. Due to the principles of noninvasive sampling, there may be some variables that cannot be measured easily, such as age, sex, real-time healthy condition or dietary shift of wild leopards, which account for the individualized bacterial microbiota at genus level. More fecal or intestinal samples from wild leopards are required for in-depth analysis of the gut bacterial community. The metabolic pathway of bacterial species should also be taken into account to provide a more comprehensive insight into the functional repertoire of the leopard gut microbiota. Although the implications of changes in gut microbiota for human and other species have been shown in many studies, the implications for wild animal conservation are still limited (Bahrndorff et al., 2016). Other studies also suggested that microbiome and fitness of host could be influenced by habitat fragmentation (Amato et al., 2013; Cheng et al., 2015). For instance, for these rare endangered animals, captivity and reintroduction into the wild are the common methods for their conservation. The gut microbiota of captive animals could be established with excepted nutritional conditions by feeding special diets as the wild individual, and reintroduction would have relative high success rate when captive animals facing uncertain environment. It is necessary to figure out the relationship between habitat fragmentation and gut microbiota, as well as the performance of diverse gut microbiota under different conditions. In general, our study presents the characterization and comparison of the gut microbiota for wild leopards, which might be able to provide a theoretical reference both for free-ranging leopards and ex-situ conservation.

## Conclusions

We first reported and compared the basic composition and structure of the fecal microbiota between wild Amur leopard and North Chinse leopard. We observed that *Firmicutes*, *Proteobacteria* and *Actinobacteria* were the three most predominant phyla in the gut microbiota of both Amur leopard and North Chinese leopard. Although their living environment and diet are relatively diverse, no significant difference was found in the main composition and structure of the gut microbiota at phylum level. We speculate that the same structure of fecal microbiota might result from genetic factors of leopard, the small sample size or too much variability within the groups. In order to understand the gut microbial ecology of Amur and North Chinese leopards, future research should focus on within-individual variation in microbial community structure, and how gut microbiome structure changes with seasonal shifts in temperature and diet. Furthermore, other methods including functional metagenomics of the gut microbiota, and whole genome sequencing of leopards, integrated with behavioural data from infrared camera traps in the field will be beneficial for leopard conservation.

##  Supplemental Information

10.7717/peerj.6684/supp-1 Figure S1 ANOSIM analysis for discrepancy of gut bacterial community between Amur leopard and North Chinese leopard *R* > 0 indicates the differences between groups were greater than it within each group. And *p* < 0.05 indicates the differences were significant.

10.7717/peerj.6684/supp-2 Figure S2 Heatmap of beta-diversity with weighted and unweighted Unifrac distances Two values in a same square represent weighted and unweighted Unifrac distances respectively, and the values are the discrepancy coefficient between two samples. There is no significant difference of gut bacterial community between Amur leopard and North Chinese leopard.

## Additional Information and Declarations

### Field Study Permissions

The following information was supplied relating to field study approvals (i.e., approving body and any reference numbers): Field experiments were approved by the Forestry Department of Jinlin Province, State Forestry Administration and Forestry Department of Shanxi Province.

### Data Availability

The following information was supplied regarding data availability: The raw data is at SRA accession number SRP149194.

## References

[ref-1] Allen GM (1938). The mammals of China and Mongolia. American Museum of Natural History.

[ref-2] Amato KR, Yeoman CJ, Kent A, Righini N, Carbonero F, Estrada A, Gaskins HR, Stumpf RM, Yildirim S, Torralba M (2013). Habitat degradation impacts black howler monkey (Alouatta pigra) gastrointestinal microbiomes. The ISME journal.

[ref-3] An C, Okamoto Y, Xu S, Eo KY, Kimura J, Yamamoto N (2017). Comparison of fecal microbiota of three captive carnivore species inhabiting Korea. Journal of Veterinary Medical Science.

[ref-4] Arumugam M, Raes J, Pelletier E, Paslier DL, Yamada T, Mende DR, Fernandes GR, Tap J, Bruls T, Batto JM (2011). Enterotypes of the human gut microbiome. Nature.

[ref-5] Bahrndorff S, Alemu T, Alemneh T, Lund Nielsen J (2016). The microbiome of animals: implications for conservation biology. International journal of genomics.

[ref-6] Balme GA, Slotow R, Hunter LT (2009). Impact of conservation interventions on the dynamics and persistence of a persecuted leopard (*Panthera pardus*) population. Biological Conservation.

[ref-7] Barelli C, Albanese D, Donati C, Pindo M, Dallago C, Rovero F, Cavalieri D, Tuohy KM, Hauffe HC, De Filippo C (2015). Habitat fragmentation is associated to gut microbiota diversity of an endangered primate: implications for conservation. Scientific Reports.

[ref-8] Barry KA, Middelbos IS, Vester Boler BM, Dowd SE, Suchodolski JS, Henrissat B, Coutinho PM, White BA, Fahey Jr G, Swanson KS (2012). Effects of dietary fiber on the feline gastrointestinal metagenome. Journal of Proteome Research.

[ref-9] Becker AA, Hesta M, Hollants J, Janssens GP, Huys G (2014). Phylogenetic analysis of faecal microbiota from captive cheetahs reveals underrepresentation of Bacteroidetes and Bifidobacteriaceae. BMC Microbiology.

[ref-10] Benson AK, Kelly SA, Legge R, Ma F, Low SJ, Kim J, Zhang M, Oh PL, Nehrenberg D, Hua K (2010). Individuality in gut microbiota composition is a complex polygenic trait shaped by multiple environmental and host genetic factors. Proceedings of the National Academy of Sciences of the United States of America.

[ref-11] Caporaso JG, Kuczynski J, Stombaugh J, Bittinger K, Bushman FD, Costello EK, Fierer N, Peña AG, Goodrich JK, Gordon JI (2010). QIIME allows analysis of high-throughput community sequencing data. Nature Methods.

[ref-12] Chang JY, Antonopoulos DA, Kalra A, Tonelli A, Khalife WT, Schmidt TM, Young VB (2008). Decreased diversity of the fecal microbiome in recurrent Clostridium difficile—associated diarrhea. The Journal of Infectious Diseases.

[ref-13] Cheng Y, Fox S, Pemberton D, Hogg C, Papenfuss AT, Belov K (2015). The Tasmanian devil microbiome—implications for conservation and management. Microbiome.

[ref-14] Clarke KR (1993). Non-parametric multivariate analyses of changes in community structure. Austral Ecology.

[ref-15] Clayton JB, Vangay P, Huang H, Ward T, Hillmann BM, Al-Ghalith GA, Travis DA, Long HT, Tuan BV, Minh VV, Cabana F, Nadler T, Toddes B, Murphy T, Glander KE, Johnson TJ, Knights D (2016). Captivity humanizes the primate microbiome. Proceedings of the National Academy of Sciences of the United States of America.

[ref-16] De FC, Cavalieri D, Di PM, Ramazzotti M, Poullet JB, Massart S, Collini S, Pieraccini G, Lionetti P (2010). Impact of diet in shaping gut microbiota revealed by a comparative study in children from Europe and rural Africa. Proceedings of the National Academy of Sciences of the United States of America.

[ref-17] Deng P, Swanson KS (2014). Gut microbiota of humans, dogs and cats: current knowledge and future opportunities and challenges. British Journal of Nutrition.

[ref-18] Dutta T, Seidensticker J (2013). Fine-scale population genetic structure in a wide-ranging carnivore, the leopard (*Panthera pardus fusca*) in central India. Diversity & Distributions.

[ref-19] Dutta T, Sharma S, Maldonado JE, Wood TC, Seidensticker J (2012). A reliable method for individual identification and gender determination of wild leopards (*Panthera pardus fusca*) using non-invasive samples. Conservation Genetics Resources.

[ref-20] Edgar RC (2004). MUSCLE: multiple sequence alignment with high accuracy and high throughput. Nucleic Acids Research.

[ref-21] Edgar RC (2013). UPARSE: highly accurate OTU sequences from microbial amplicon reads. Nature Methods.

[ref-22] Edgar RC, Haas BJ, Clemente JC, Quince C, Knight R (2011). UCHIME improves sensitivity and speed of chimera detection. Bioinformatics.

[ref-23] Evans NJ, Brown JM, Murray RD, Brian G, Birtles RJ, Anthony HC, Carter SD (2011). Characterization of novel bovine gastrointestinal tract Treponema isolates and comparison with bovine digital dermatitis treponemes. Applied and Environmental Microbiolgy.

[ref-24] Fang W, Fang Z, Zhou P, Chang F, Hong Y, Zhang X, Peng H, Xiao Y (2012). Evidence for lignin oxidation by the giant panda fecal microbiome. PLOS ONE.

[ref-25] Feng L, Shevtsova E, Vitkalova A, Matyukhina D, Miquelle D, Aramilev V, Wang T, Mu P, Xu R, Ge J (2017). Collaboration brings hope for the last Amur leopards. Cat News.

[ref-26] Garcia-Mazcorro JF, Dowd SE, Jeffrey P, Steiner JM, Suchodolski JS (2012). Abundance and short-term temporal variability of fecal microbiota in healthy dogs. Microbiologyopen.

[ref-27] Garciamazcorro JF, Lanerie DJ, Dowd SE, Paddock CG, Grützner N, Steiner JM, Ivanek R, Suchodolski JS (2011). Effect of a multi-species synbiotic formulation on fecal bacterial microbiota of healthy cats and dogs as evaluated by pyrosequencing. Fems Microbiology Ecology.

[ref-28] Gibson GR, Probert HM, Loo JV, Rastall RA, Roberfroid MB (1995). Dietary modulation of the human colonic microbiota: updating the concept of prebiotics. Journal of Nutrition.

[ref-29] Gomez A, Petrzelkova K, Yeoman CJ, Vlckova K, Koppova I, Carbonero F, Ulanov A, Modry D, Todd A, Torralba M (2015). Gut microbiome composition and metabolomic profiles of wild western lowland gorillas (Gorilla gorilla gorilla) reflect host ecology. Molecular Ecology.

[ref-30] Gray JE (1862). Description of some New Species of Mammalia.

[ref-31] Guan Y, Yang H, Han S, Feng L, Wang T, Ge J (2017). Comparison of the gut microbiota composition between wild and captive sika deer (*Cervus nippon hortulorum*) from feces by high-throughput sequencing. AMB Express.

[ref-32] Haas BJ, Gevers D, Earl AM, Feldgarden M, Ward DV, Giannoukos G, Ciulla D, Tabbaa D, Highlander SK, Sodergren E (2011). Chimeric 16S rRNA sequence formation and detection in Sanger and 454-pyrosequenced PCR amplicons. Genome Research.

[ref-33] Handl S, Dowd SE, Garciamazcorro JF, Steiner JM, Suchodolski JS (2011). Massive parallel 16S rRNA gene pyrosequencing reveals highly diverse fecal bacterial and fungal communities in healthy dogs and cats. Fems Microbiology Ecology.

[ref-34] Harri M, Jukka C, Lars P, Anna K, Lotta KK, Jarno T, Airi P (2009). Sequence analysis of percent G+C fraction libraries of human faecal bacterial DNA reveals a high number of Actinobacteria. BMC Microbiology.

[ref-35] Hebblewhite M, Miquelle DG, Murzin AA, Aramilev VV, Pikunov DG (2011). Predicting potential habitat and population size for reintroduction of the Far Eastern leopards in the Russian Far East. Biological Conservation.

[ref-36] Hentges DJ, Hentges DJ (1983). Role of the intestinal microflora in host defense against infection. Human intestinal microflora in health and disease.

[ref-37] Hildebrandt MA, Hoffmann C, Sherrillmix SA, Keilbaugh SA, Hamady M, Chen YY, Knight R, Ahima RS, Bushman F, Wu GD (2009). High-fat diet determines the composition of the murine gut microbiome independently of obesity. Gastroenterology.

[ref-38] Jackson P, Nowell K (2008). Panthera pardus ssp. orientalis. The IUCN Red List of Threatened Species 2008. RLTS T15957A5333757 en.

[ref-39] Jacobson AP, Gerngross P, Lemeris Jr J, Schoonover RF, Anco C, Breitenmoser-Würsten C, Durant SM, Farhadinia MS, Henschel P, Kamler JF (2016). Leopard (*Panthera pardus*) status, distribution, and the research efforts across its range. Peerj.

[ref-40] Johnston KL, Swift NC, Forster-van Hijfte M, Rutgers HC, Lamport A, Ballàvre O, Batt RM (2001). Comparison of the bacterial flora of the duodenum in healthy cats and cats with signs of gastrointestinal tract disease. Journal of the American Veterinary Medical Association.

[ref-41] Kameyama K, Itoh K (2014). Intestinal colonization by a Lachnospiraceae bacterium contributes to the development of diabetes in obese mice. Microbes and Environments.

[ref-42] Kissui BM (2008). Livestock predation by lions, leopards, spotted hyenas, and their vulnerability to retaliatory killing in the Maasai steppe, Tanzania. Animal Conservation.

[ref-43] Kitchener AC, Breitenmoser C, Eizirik E, Gentry A, Werdelin L, Wilting A, Yamaguchi N, Abramov AV, Christiansen P, Driscoll CA, Duckworth W, Johnson WE, Luo S, Meijaard E, Donoghue PO, Sanderson J, Seymour K, Bruford MW, Groves C, Hoffmann M, Nowell K, Timmons Z, Tobe SS (2017). A revised taxonomy of the Felidae. Cat News.

[ref-44] Lan PT, Sakamoto M, Sakata S, Benno Y (2006). *Bacteroides barnesiae* sp. nov. *Bacteroides salanitronis* sp. nov. and *Bacteroides gallinarum* sp. nov. isolated from chicken caecum. International Journal of Systematic & Evolutionary Microbiology.

[ref-45] Ley RE, Bäckhed F, Turnbaugh P, Lozupone CA, Knight RD, Gordon JI (2005). Obesity alters gut microbial ecology. Proceedings of the National Academy of Sciences of the United States of America.

[ref-46] Ley RE, Hamady M, Lozupone C, Turnbaugh PJ, Ramey RR, Bircher JS, Schlegel ML, Tucker TA, Schrenzel MD, Knight R (2008). Evolution of mammals and their gut microbes. Science.

[ref-47] Ley RE, Peterson DA, Gordon JI (2006). Ecological and evolutionary forces shaping microbial diversity in the human intestine. Cell.

[ref-48] Lubbs DC, Vester BM, Fastinger ND, Swanson KS (2009). Dietary protein concentration affects intestinal microbiota of adult cats: a study using DGGE and qPCR to evaluate differences in microbial populations in the feline gastrointestinal tract. Journal of Animal Physiology and Animal Nutrition.

[ref-49] Martin M (2011). Cutadapt removes adapter sequences from high-throughput sequencing reads. EMBnet journal.

[ref-50] Martins Q, Horsnell W, Titus W, Rautenbach T, Harris S (2011). Diet determination of the Cape Mountain leopards using global positioning system location clusters and scat analysis. Journal of Zoology.

[ref-51] Maynard CL, Elson CO, Hatton RD, Weaver CT (2012). Reciprocal interactions of the intestinal microbiota and immune system. Nature.

[ref-52] Meehan CJ, Beiko RG (2014). A phylogenomic view of ecological specialization in the Lachnospiraceae, a family of digestive tract-associated bacteria. Genome Biology and Evolution.

[ref-53] Middelbos IS, Boler BMV, Qu A, White BA, Swanson KS, Fahey GC (2010). Phylogenetic characterization of fecal microbial communities of dogs fed diets with or without supplemental dietary fibery using 454 pyrosequencing. PLOS ONE.

[ref-54] Miththapala S, Seidensticker J, O’Brien SJ (1996). Phylogeographic subspecies recognition in leopards (*Panthera pardus*): molecular genetic variation. Conservation Biology.

[ref-55] Musso G, Gambino R, Cassader M (2010). Gut microbiota as a regulator of energy homeostasis and ectopic fat deposition: mechanisms and implications for metabolic disorders. Current Opinion in Lipidology.

[ref-56] Nelson TM, Rogers TL, Carlini AR, Brown MV (2013). Diet and phylogeny shape the gut microbiota of Antarctic seals: a comparison of wild and captive animals. Environmental Microbiology.

[ref-57] Nicholson JK, Holmes E, Kinross J, Burcelin R, Gibson G, Jia W, Pettersson S (2012). Host-gut microbiota metabolic interactions. Science.

[ref-58] Nowell K, Jackson P (1996). Status survey and conservation action plan: wild cats. Biological Conservation.

[ref-59] Ozaki Y, Diwan AR, Mcbride WD, Melnick JL (2009). Characterization and quantification of feline fecal microbiota using cpn60 sequence-based methods and investigation of animal-to-animal variation in microbial population structure. Veterinary Microbiology.

[ref-60] Packer C, Brink H, Kissui BM, Maliti H, Kushnir H, Caro T (2011). Effects of trophy hunting on lion and leopard populations in Tanzania. Conservation Biology.

[ref-61] Packey CD, Sartor RB (2009). Commensal bacteria, traditional and opportunistic pathogens, dysbiosis and bacterial killing in inflammatory bowel diseases. Current Opinion in Infectious Diseases.

[ref-62] Park HJ, Lee SE, Kim HB, Isaacson R, Seo KW, Song KH (2015). Association of obesity with serum leptin, adiponectin, and serotonin and gut microflora in beagle dogs. Journal of Veterinary Internal Medicine.

[ref-63] Quast C, Pruesse E, Yilmaz P, Gerken J, Schweer T, Yarza P, Peplies J, Glöckner FO (2013). The SILVA ribosomal RNA gene database project: improved data processing and web-based tools. Nucleic Acids Research.

[ref-64] Queen EV, Marks SL, Farver TB (2012). Prevalence of selected bacterial and parasitic agents in feces from diarrheic and healthy control cats from Northern California. Journal of Veterinary Internal Medicine.

[ref-65] R Core Team (2013). https://www.R-project.org/.

[ref-66] Reeves AR, Wang GR, Salyers AA (1997). Characterization of four outer membrane proteins that play a role in utilization of starch by Bacteroides thetaiotaomicron. Journal of Bacteriology.

[ref-67] Ritchie LE, Burke KF, Garcia-Mazcorro JF, Steiner JM, Suchodolski JS (2010). Characterization of fecal microbiota in cats using universal 16S rRNA gene and group-specific primers for Lactobacillus and Bifidobacterium spp. Veterinary Microbiology.

[ref-68] Ritchie LE, Steiner JM, Suchodolski JS (2008). Assessment of microbial diversity along the feline intestinal tract using 16S rRNA gene analysis. Fems Microbiology Ecology.

[ref-69] Rodgers TW, Janečka JE (2013). Applications and techniques for non-invasive faecal genetics research in felid conservation. European Journal of Wildlife Research.

[ref-70] Round JL, Mazmanian SK (2009). The gut microbiota shapes intestinal immune responses during health and disease. Nature Reviews Immunology.

[ref-71] Schloss PD, Westcott SL, Ryabin T, Hall JR, Hartmann M, Hollister EB, Lesniewski RA, Oakley BB, Parks DH, Robinson CJ (2009). Introducing mothur: open-source, platform-independent, community-supported software for describing and domparing microbial cmmunities. Applied & Environmental Microbiology.

[ref-72] Schwab C, Cristescu B, Northrup JM, Stenhouse GB, Gänzle M (2011). Diet and environment shape fecal bacterial microbiota composition and enteric pathogen load of grizzly bears. PLOS ONE.

[ref-73] Shen F, Zheng R, Sun X, Ding W, Wang X, Fan J (2017). Gut microbiota dysbiosis in patients with non-alcoholic fatty liver disease. Hepatobiliary & Pancreatic Diseases International.

[ref-74] Sommer F, Bäckhed F (2013). The gut microbiota- masters of host development and physiology. Nature Reviews Microbiology.

[ref-75] Stein A, Athreya V, Gerngross P, Balme G, Henschel P, Karanth U, Miquelle D, Rostro S, Kamler J, Laguardia A (2016). Panthera pardus. The IUCN Red List of Threatened Species 2016: e. T15954A50659089 Gland: IUCN.

[ref-76] Suchodolski JS (2011). Companion animals symposium: microbes and gastrointestinal health of dogs and cats. Journal of Animal Science.

[ref-77] Suchodolski JS (2016). Diagnosis and interpretation of intestinal dysbiosis in dogs and cats. Veterinary Journal.

[ref-78] Suchodolski JS, Foster ML, Sohail MU, Leutenegger C, Queen EV, Steiner JM, Marks SL (2015). The fecal microbiome in cats with diarrhea. PLOS ONE.

[ref-79] Suchodolski JS, Markel ME, Garcia-Mazcorro JF, Unterer S, Heilmann RM, Dowd SE, Kachroo P, Ivanov I, Minamoto Y, Dillman EM, Steiner JM, Cook AK, Toresson L (2012). The fecal microbiome in dogs with acute diarrhea and idiopathic inflammatory bowel disease. PLOS ONE.

[ref-80] Sunquist M, Sunquist F (2002). Wild cats of the world.

[ref-81] Tian Y, Wu J, Smith AT, Wang T, Kou X, Ge J (2015). Population viability of the Siberian Tiger in a changing landscape: Going, going and gone?. Ecological Modelling.

[ref-82] Tremaroli V, Bäckhed F (2012). Functional interactions between the gut microbiota and host metabolism. Nature.

[ref-83] Tun HM, Brar MS, Khin N, Jun L, Hui RK, Dowd SE, Leung FC (2012). Gene-centric metagenomics analysis of feline intestinal microbiome using 454 junior pyrosequencing. Journal of Microbiological Methods.

[ref-84] Turnbaugh PJ, Bäckhed F, Fulton L, Gordon JI (2008). Diet-induced obesity is linked to marked but reversible alterations in the mouse distal gut microbiome. Cell Host & Microbe.

[ref-85] Turnbaugh PJ, Ley RE, Mahowald MA, Magrini V, Mardis ER, Gordon JI (2006). An obesity-associated gut microbiome with increased capacity for energy harvest. Nature.

[ref-86] Uphyrkina O, Johnson WE, Quigley H, Miquelle D, Marker L, Bush M, O’Brien SJ (2001). Phylogenetics, genome diversity and origin of modern leopard, Panthera pardus. Molecular Ecology.

[ref-87] Van der Waaij D, Berghuis-de Vries J, Lekkerkerk-Van der Wees J (1971). Colonization resistance of the digestive tract in conventional and antibiotic-treated mice. Epidemiology & Infection.

[ref-88] Wang Q, Garrity GM, Tiedje JM, Cole JR (2007). Naive Bayesian classifier for rapid assignment of rRNA sequences into the new bacterial taxonomy. Applied & Environmental Microbiology.

[ref-89] Wang T, Feng L, Yang H, Han B, Zhao Y, Juan L, Lü X, Zou L, Li T, Xiao W (2017). A science-based approach to guide Amur leopard recovery in China. Biological Conservation.

[ref-90] Wu H, Wan QH, Fang SG (2004). Two genetically distinct units of the Chinese sika deer (*cervus nippon*): analyses of mitochondrial DNA variation. Biological Conservation.

[ref-91] Wu X, Zhang H, Chen J, Shang S, Yan J, Chen Y, Tang X, Zhang H (2017). Analysis and comparison of the wolf microbiome under different environmental factors using three different data of Next Generation Sequencing. Scientific Reports.

[ref-92] Yang H, Dou H, Baniya RK, Han S, Guan Y, Xie B, Zhao G, Wang T, Mou P, Feng L, Ge J (2018). Seasonal food habits and prey selection of Amur tigers and Amur leopards in Northeast China. Scientific Reports.

[ref-93] Yasushi M, Otoni CC, Steelman SM, Olga B, Steiner JRM, Jergens AE, Suchodolski JS (2015). Alteration of the fecal microbiota and serum metabolite profiles in dogs with idiopathic inflammatory bowel disease. Gut Microbes.

[ref-94] Zhang H, Chen L (2010). Phylogenetic analysis of 16S rRNA gene sequences reveals distal gut bacterial diversity in wild wolves (*Canis lupus*). Molecular Biology Reports.

[ref-95] Zhang H, Liu G, Chen L, Sha W (2015). Composition and diversity of the bacterial community in snow leopard (*Uncia uncia*) distal gut. Annals of Microbiology.

[ref-96] Zhao N, Li M, Luo J, Wang S, Liu S, Wang S, Lyu W, Chen L, Su W, Ding H (2017). Impacts of canine distemper virus infection on the giant panda population from the perspective of gut microbiota. Scientific Reports.

[ref-97] Zheng J, Zhang Y, Wang Y, Dong D (2009). The characteristics of plant distribution and diversity in the middle section of Taihang Mountain. Henan Science.

